# Luciferase activity under direct ligand-dependent control of a muscarinic acetylcholine receptor

**DOI:** 10.1186/1472-6750-9-46

**Published:** 2009-05-18

**Authors:** Doreen Thor, Diana Le Duc, Rainer Strotmann, Torsten Schöneberg

**Affiliations:** 1 Department of Molecular Biochemistry, Institute of Biochemistry, Medical Faculty, University of Leipzig, Johannisallee 30, 04103 Leipzig, Germany

## Abstract

**Background:**

Controlling enzyme activity by ligand binding to a regulatory domain of choice may have many applications e.g. as biosensors and as tools in regulating cellular functions. However, until now only a small number of ligand-binding domains have been successfully linked to enzyme activity. G protein-coupled receptors (GPCR) are capable of recognizing an extraordinary structural variety of extracellular signals including inorganic and organic molecules. Ligand binding to GPCR results in conformational changes involving the transmembrane helices. Here, we assessed whether ligand-induced conformational changes within the GPCR helix bundle can be utilized to control the activity of an integrated enzyme.

**Results:**

As a proof of principle, we inserted the luciferase amino acid sequence into the third intracellular loop of the M_3 _muscarinic acetylcholine receptor. This fusion protein retained both receptor and enzyme function. Receptor blockers slightly but significantly reduced enzyme activity. By successive deletion mutagenesis the enzyme activity was optimally coupled to ligand-induced conformational helix movements.

**Conclusion:**

Our results demonstrate that in engineered GPCR-enzyme chimeras, intracellular enzyme activity can be directly controlled by a GPCR serving as the extracellular ligand-binding domain.

## Background

Synthetic protein biosensors are typically designed by fusing a target-binding domain to an easily assayed reporter protein. The ligand-binding domain is often derived from a specific receptor protein. Most biosensors are complementation systems where enzyme or fluorophore activity is reconstituted from non-functional domains secondary to receptor dimerization or conformational changes upon ligand binding [1,2]. However, the repertoires of both, well characterized ligand-binding and reporter domains that are suitable for the design of complementation systems are rather small. A modular system that allows arbitrary combination of ligand-binding and reporter domains would thus be most desirable. There are a few examples of properly constructed receptor-enzyme chimeras which allow for the transduction of ligand-induced conformational changes within the receptor to the reporter protein and allosteric modulation of its properties [3-5]. Coupling of ligand-induced conformational changes to reporter protein function appears to be the general bottleneck in designing such biosensors.

Among the different families of transmembrane receptors, G protein-coupled receptors (GPCR) form the largest receptor superfamily comprising over 1000 members within several vertebrate genomes [6-8]. Signals as multiform as light, small molecules including ions, amines, amino acids, peptides, lipids, sugars, as well as large proteins are recognized by receptors of this class [9,10]. Upon agonist binding to the extracellular portion of GPCR, conformational changes of the transmembrane helix (TMH) bundle and intracellular loop (ICL) portions lead to G-protein activation [11-16]. Conformational changes of the TMH bundle are not only observed after agonist binding but also after binding of inverse agonists that do not result in G-protein activation [17-19]. In addition, GPCR can be modified by site-directed mutagenesis to respond to biologically inert compounds instead of their native agonists [20,21]. The combination of these properties favours GPCR as ideal ligand-binding modules in hybrid biosensors.

In a number of studies, enzymes, such as luciferases, galactosidase, alkaline phosphatase and peroxidase, as well as fluorescent proteins (e.g. YFP) have been integrated into GPCR. In these GPCR fusion proteins, the enzyme or fluorescence activities were used as reporter assay to monitor intracellular receptor trafficking [22,23] and, in fluorescence and bioluminescence resonance energy transfer (FRET/BRET) approaches, to monitor GPCR-protein interactions [24-27]. Recently, the activity of ion channels coupled to GPCR was modulated upon ligand binding, demonstrating that GPCR are suitable binding domains for biosensors [28].

Here, we report the allosteric modulation of enzyme activity upon ligand binding to a GPCR-enzyme chimera. Using the M_3 _muscarinic acetylcholine receptor (M_3_R) and luciferase as model proteins, we provide a proof of concept that through rigid-body movement of the TMH bundle, extracellular signals may be transduced onto an enzyme that is integrated into the cytosolic portion of a GPCR, thus changing enzyme activity.

## Results and discussion

### Functional integration of luciferase into the M_3_R

**Figure 1:**
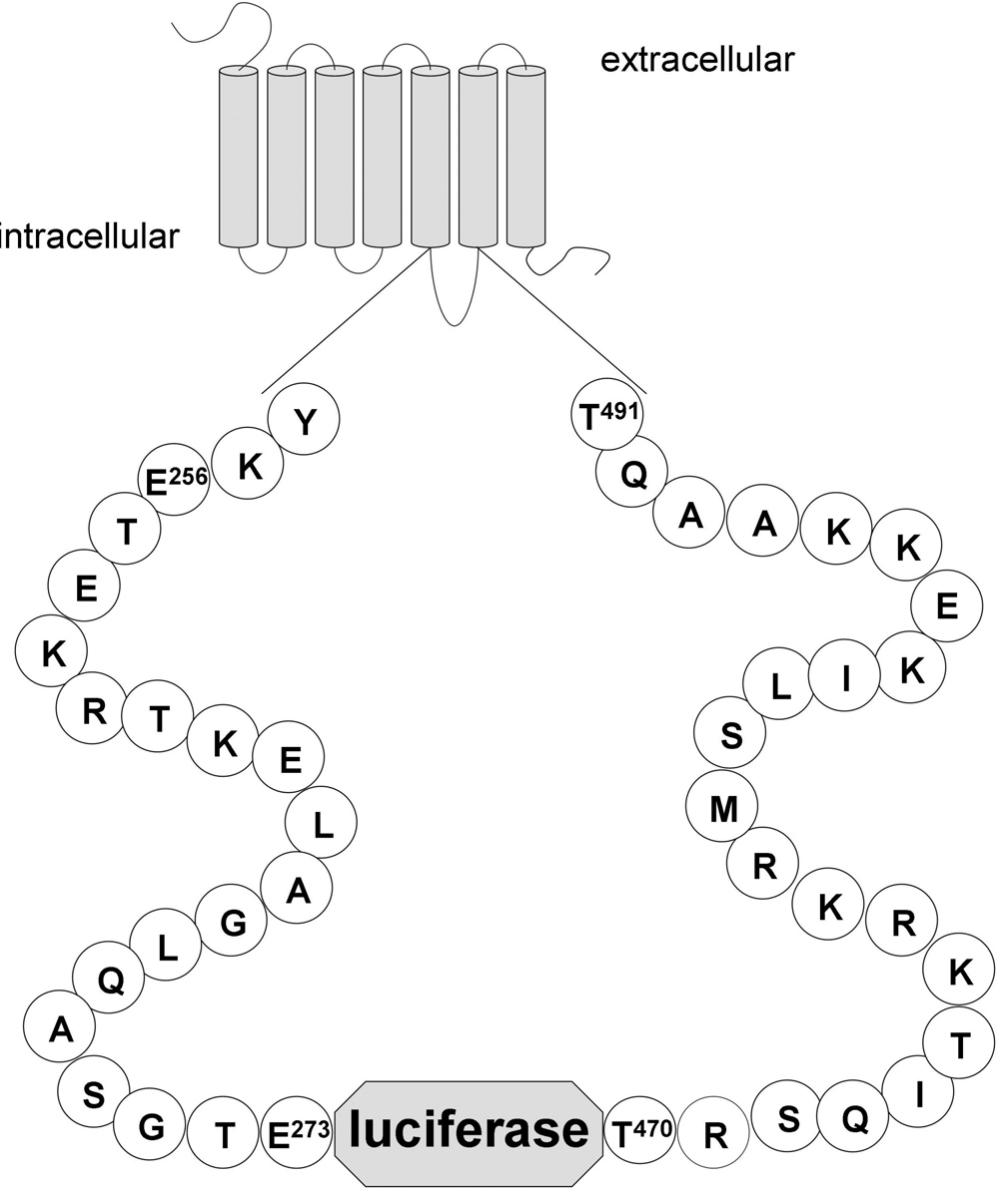
**Schematic presentation of the M_3_R-luciferase fusion construct**. Outline of M_3_R-luci with detailed illustration of ICL3 containing the luciferase sequence. Details of all fusion constructs are given in Figure 2.

**Figure 2:**
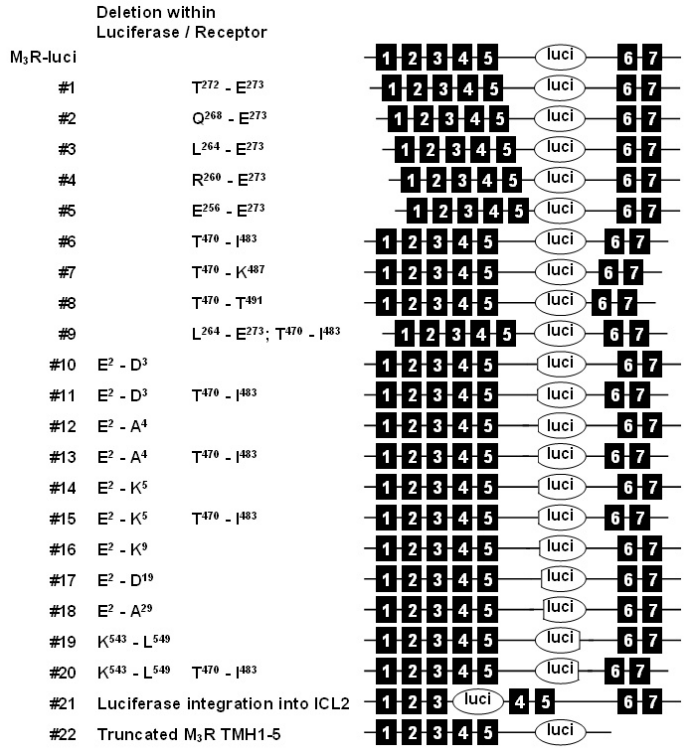
**Fusion constructs and deletion mutants used in the study**. Mutants were generated by successive deletion of N- and C-terminal domains of the enzyme and loop portions of the receptor (deleted portions are given). For control experiments luciferase was also integrated into ICL2 (#21) and fused to a truncated M_3_R only consisting of TMH1-5 (#22).

**Figure 3:**
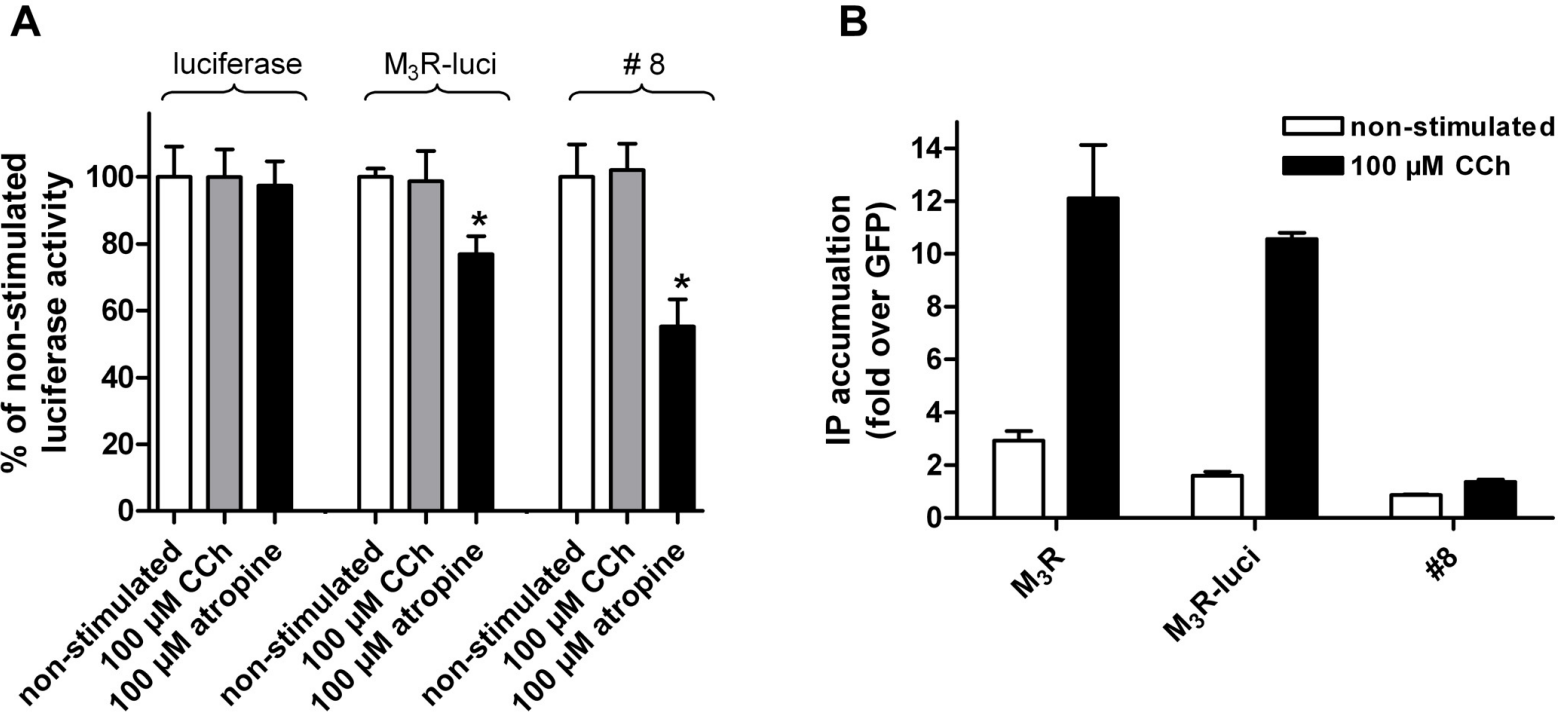
**Functional integration of luciferase into the M_3 _muscarinic acetylcholine receptor**. A) COS-7 cells were transfected with luciferase construct, M_3_R-luci and construct #8 and luciferase activity was determined after incubation without and with the indicated ligands. The luminescences without ligands were 1,905,212 ± 172,463 AU (luciferase), 196,512 ± 4,942 AU (M_3_R-luci) and 100,473 ± 9,770 AU (#8). All data are given as means ± SEM of three independent experiments each performed in triplicate. B) Basal and CCh-induced IP formation was determined in COS-7 cells transfected with the wild-type M_3_R, M_3_R-luci and construct #8. All data are given as means ± SEM of three independent experiments each performed in triplicate.

To generate a biosensor in which enzyme activity is allosterically controlled by a binding domain of a GPCR, we replaced a 195-amino acid segment of the third ICL (ICL3) of M_3_R (amino acid positions 274–469) with the sequence of luciferase from *Photinus pyralis *(referred to as M_3_R-luci, Figures 1 and 2). Previous studies in mammalian expression systems have shown that in M_3_R, the removal of the central part of ICL3 has no significant effect on receptor function [29-31]. As shown in Figure 3 and summarized in Table 1, M_3_R-luci expressed in COS-7 cells was delivered to the cell surface and displayed both luciferase activity (Figure 3A) and carbachol (CCh)-induced inositol phosphate (IP) formation (Figure 3B). However, enzyme activity was not influenced by application of the agonist CCh but we noted a significant reduction (15%, p < 0.001) in luciferase activity in the presence of the inverse agonist atropine (Figure 3A, Table 1). The effect of atropine was not found when luciferase was inserted into the second intracellular loop (ICL2) or fused to a truncated M_3_R (constructs #21 and #22, Figure 2, Table 1) and in several other controls (luciferase alone, V_2 _vasopressin receptor (V_2_R)) [see Additional file 1] [see Additional file 2] [see Additional file 3].

**Table 1:** Functional properties of wild-type M_3_R and M_3_R-luciferase fusion proteins.

	luciferase activity			IP accumulation (fold over GFP basal)		Cell surface expression
mutant	basal (% of M_3_R-luci)	100 μM CCh (% of basal activity)	100 μM atropine (% of basal activity)	basal	100 μM CCh	(% of M_3_R-luci)
M_3_R	0.08 ± 0.03 (3)	-	-	2.93 ± 0.37	12.1 ± 2.0	229 ± 13
M_3_R-luci	100 (31)	105 ± 5	84.6 ± 3.5**	1.60 ± 0.15	10.6 ± 0.2	100
#1	71.2 ± 0.7 (3)	103 ± 9	74.8 ± 9.3	1.40 ± 0.12	9.60 ± 0.45	74.7 ± 5.3
#2	42.8 ± 4.8 (3)	116 ± 5	93.0 ± 4.2	1.20 ± 0.12	4.37 ± 0.09	56.1 ± 4.1
#3	29.8 ± 3.0 (4)	91.0 ± 11.0	95.2 ± 10.9	0.93 ± 0.03	1.57 ± 0.12	32.8 ± 5.3
#4	16.6 ± 3.0 (3)	110 ± 3.8	104 ± 6.8	0.97 ± 0.03	1.10 ± 0.06	13.5 ± 0.7
#5	11.1 ± 1.5 (3)	104 ± 10	96.0 ± 21.2	0.97 ± 0.09	1.10 ± 0.06	3.9 ± 0.9
#6	95.7 ± 5.5 (20)	94.0 ± 7.1	75.7 ± 5.2**	1.27 ± 0.09	9.50 ± 0.61	105 ± 3.2
#7	42.0 ± 3.7 (11)	95.9 ± 7.5	93.8 ± 5.9	1.00 ± 0.10	3.27 ± 0.17	35.8 ± 4.2
#8	62.6 ± 5.2 (22)	98.5 ± 4.7	60.2 ± 2.8**	0.87 ± 0.03	1.37 ± 0.09	114 ± 7
#9	25.9 ± 3.7 (5)	98.4 ± 8.7	87.3 ± 8.6	1.03 ± 0.09	1.43 ± 0.12	27.3 ± 3.5
#10	37.0 ± 4.0 (5)	95.2 ± 7.3	76.4 ± 4.1*	1.53 ± 0.17	9.67 ± 0.78	31.6 ± 4.4
#11	30.3 ± 3.9 (5)	83.4 ± 7.3	73.8 ± 3.1*	1.40 ± 0.06	8.33 ± 0.54	28.9 ± 4.8
#12	37.2 ± 6.8 (4)	111 ± 17	78.6 ± 10.1	1.43 ± 0.17	9.90 ± 0.70	56.6 ± 7.8
#13	27.8 ± 6.7 (3)	80.2 ± 8.3	76.3 ± 8.5	1.13 ± 0.09	8.87 ± 1.01	36.7 ± 11.6
#14	42.0 ± 11.3 (4)	106 ± 10	85.5 ± 6.4	1.50 ± 0.12	9.57 ± 0.95	39.5 ± 4.3
#15	31.8 ± 6.5 (4)	102 ± 5	84.3 ± 7.4	1.10 ± 0.10	8.80 ± 0.95	25.5 ± 2.5
#16	0.4 ± 0.1 (3)	-	-	1.07 ± 0.09	5.23 ± 0.41	14.6 ± 3.8
#17	0.4 ± 0.1 (3)	-	-	1.00 ± 0.06	4.40 ± 0.45	12.3 ± 5.7
#18	0.5 ± 0.3 (3)	-	-	0.87 ± 0.07	1.80 ± 0.15	5.9 ± 2.5
#19	69.3 ± 7.6 (4)	92.3 ± 16.1	66.4 ± 9.4	1.47 ± 0.19	10.9 ± 1.5	76.6 ± 12.6
#20	19.5 ± 1.3 (4)	99.6 ± 4.5	91.8 ± 9.0	0.93 ± 0.09	4.13 ± 0.12	35.2 ± 2.9
#21	17.8 ± 2.4 (7)	99.5 ± 3.3	96.1 ± 4.1	1.07 ± 0.19	1.03 ± 0.07	6.6 ± 1.6
#22	24.3 ± 10.9 (5)	97.4 ± 4.2	97.2 ± 9.1	1.03 ± 0.07	0.93 ± 0.09	0.6 ± 0.5

To evaluate the functional properties of M_3_R-enzyme chimeras, luciferase activities, measured in the absence (basal activity) and presence of the indicated ligands, were determined (see Methods). Basal activity is given as percentage of M_3_R-luci (411,043 ± 95,142 AU). Activity in the presence of ligands is given as percentage of the activity of the individual constructs. Data are presented as means ± S.E.M. of the indicated number of experiments, each carried out in triplicate. For measurement of G_q_/phospholipase C activation IP accumulation assays were performed as described under Methods. Basal IP values were determined in control (GFP)-transfected COS-7 cells (716 ± 81 cpm/well). Data (three independent assays performed in triplicate) are given as means ± S.E.M. fold over GFP-transfected IP values. Cell surface expression was assayed with indirect cell surface ELISA. Specific optical density (OD) readings (OD value of HA-tagged construct minus OD value of GFP-transfected cells) are given as a percentage of M_3_R-luci. The nonspecific OD (GFP) was 0.156 ± 0.02 (set as 0%) and the OD value of M_3_R-luci was 0.66 ± 0.06 (set as 100%). ELISA data are given as means ± S.E.M. of three independent experiments, each carried out in triplicate.

Significant reduction of the luciferase activity (*p < 0.01; **p < 0.001)

### Optimization of the M_3_R-luciferase fusion protein

Next, we systematically deleted portions of ICL3 flanking the enzyme (Figure 2) to improve coupling of atropine-induced conformational changes to enzyme activity. Successive N-terminal shortening of ICL3 (constructs #1-#5) progressively reduced cell surface expression, ligand-induced IP formation and luciferase activity but atropine failed to modulate luciferase activity. Likewise, the C-terminal part of ICL3 was shortened (constructs #6-#8). Constructs #6 and #8 were properly delivered to the cell surface and displayed high luciferase activity. Strikingly, both chimeras showed a significant reduction in enzyme activity up to ~40% (construct #8, Table 1) upon atropine binding. G-protein signalling was retained in construct #6 but abolished in construct #8, presumably because the deleted C-terminal part of ICL3 is involved in G-protein coupling [32]. The combination of N- and C-terminal shortening (construct #9) was not advantageous.

We then tested whether N- or C-terminal truncation of the luciferase insert improved coupling to conformational changes of the receptor. Only deletion of the very N-terminal two amino acid residues (construct #10) had a significant effect on atropine-induced reduction of the enzyme activity (Table 1). Combination with ICL3 shortening (construct #11) did not further improve allosteric modulation of the enzyme activity by atropine. In agreement with previous studies [33], further removal of the N terminus abolished enzyme activity completely (Table 1, constructs #12-#18) probably because of destruction of the functionally relevant N-terminal domain. The crystal structure of *Photinys pyralis *luciferase demonstrates that N-terminal and C-terminal domains form the active site [34]. C-terminal truncation of the enzyme did not result in significantly reduced enzyme activity after atropine application, neither alone (construct #19) nor in combination with ICL3 truncation (construct #20). Preliminary studies with fusion proteins of other GPCR and reporter proteins indicate that integration and optimization is required for every individual biosensor (data not shown).

### Allosteric modulation of luciferase activity by M_3_R blockers

**Figure 4:**
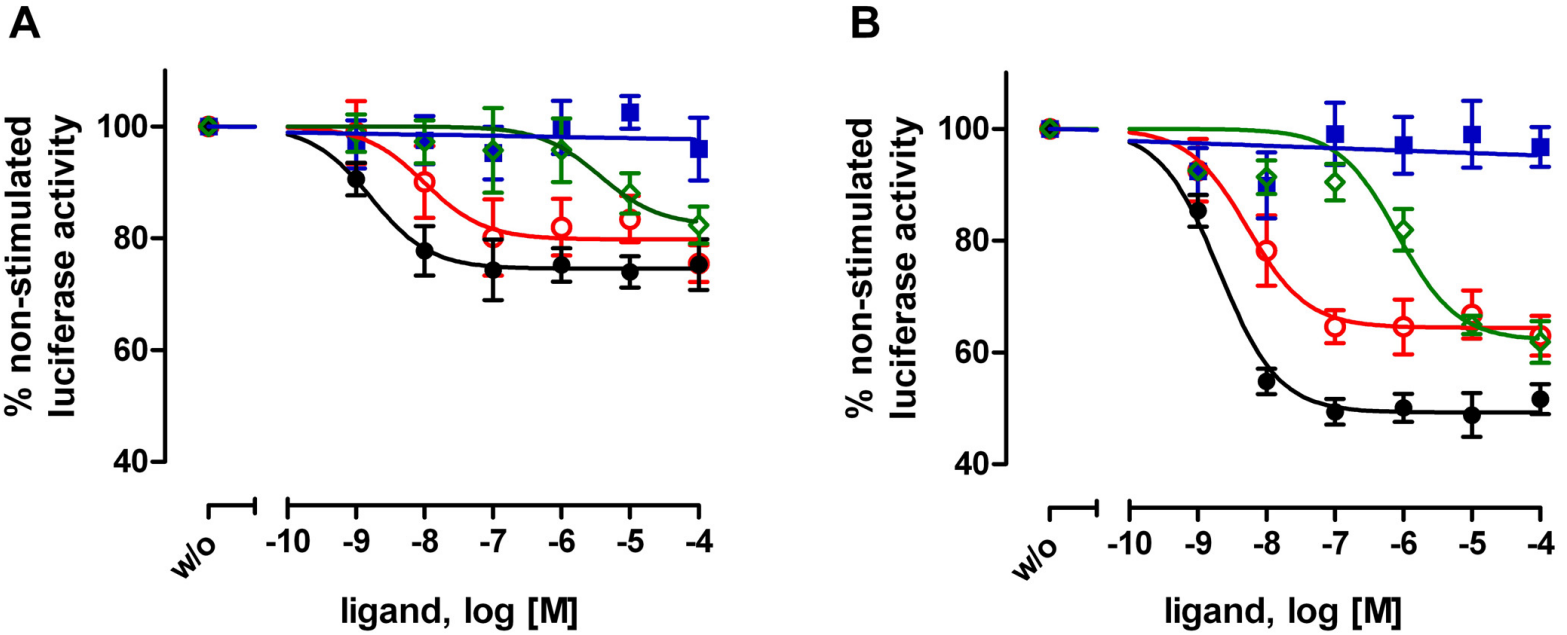
**Allosteric modulation of enzyme activity in M_3_R-luciferase fusion proteins by nanomolar concentrations of M_3_R blockers**. CCh (square), atropine (open circle), scopolamine (filled circle) and butylscopolamine (diamond) were applied at the indicated concentrations on COS-7 cells transfected with M_3_R-luci (A) and construct #8 (B) and luciferase activity was determined. The luminescences without ligands were 178,680 ± 18,171 AU (M_3_R-luci) and 121,578 ± 12,845 AU (construct #8). Enzyme activity of the individual constructs without ligands was set at 100%. All data are given as mean ± S.E.M. of four independent experiments each performed in triplicate. Control experiments were carried out with COS-7 cells expressing luciferase alone and V_2_R-luci [see Additional file 3].

Finally, we investigated the specificity and potency of other M_3_R blockers in M_3_R-luci constructs. Scopolamine and butylscopolamine, both are inverse agonists at M_3_R, were most efficient in reducing luciferase activity in construct #8 [see Additional file 4]. IC_50 _values for atropine and scopolamine were 6.6 ± 2.3 nM and 2.1 ± 0.4 nM, respectively (Figure 4). In agreement with functional studies at the wild-type M_3_R [35], butylscopolamine was less potent in luciferase inhibition (IC_50 _value: 1.7 ± 0.5 μM, Figure 4). This indicates an unchanged pharmacology of the ligand-binding domain within the M_3_R-luciferase fusion protein.

From the constructs tested, construct #8 met best the criteria of a biosensor, including nanomolar ligand sensitivity, high cell surface expression, and reduced G protein-coupling (Figures 3 and 4, Table 1). In this construct, structural optimization was successfully used to enhance coupling of ligand-induced rigid body movement of the TMH to structural changes within the luciferase domain. As this leads to a reduction in luciferase activity, one can speculate that the underlying conformational change rearranges the orientation of the N- and C-terminal domains within the luciferase molecule that form its active centre. In addition, our data support previous findings [18,19] that structural changes upon inverse agonist binding are different from those induced by agonists. Following CCh stimulation, fusion protein #6 still activated the G_q_/phospholipase C pathway but CCh had no influence on luciferase activity (Table 1). However, atropine reduced luciferase activity by 25% in this construct. It is interesting to note that insertion of luciferase, which itself is bigger than the receptor protein, into the cytoplasmic surface of the GPCR still allows for efficient G-protein coupling. This implicates a rather small interaction site between the receptor and the G protein.

## Conclusion

By integration of luciferase into ICL3 of a GPCR and successive optimization, we generated a fusion protein in which the enzyme activity is allosterically modulated by ligand binding to the GPCR in the nanomolar range. Thus, we demonstrate that GPCR are suitable as modular ligand binding domains capable of transducing the signal through rigid-body movement of TMH onto an intracellularly integrated enzyme. Artificial GPCR-enzyme chimeras may have applications beyond biosensing, for example, in ligand-dependent control of metabolic pathways and as molecular tools to study conformational changes in GPCR. Our study may also encourage the generation of GPCR fusion proteins in which other proteins or protein domains such as fluorescent proteins and SH3 domains are allosterically regulated to modulate fluorescence properties and protein-protein interactions, respectively.

## Methods

### Materials

The agonist carbamylcholine chloride (carbachol, CCh) and the inverse agonists [36] atropine sulfate, scopolamine hydrochloride, and n-butyl scopolamine bromide were obtained from Sigma. Substances were solved in water and stock solutions (100 mM) were aliquoted and stored at -20°C. Aliquots were thawed only once. All restriction enzymes for cloning purposes were purchased from NEB and primers [see Additional file 5] were synthesized by Invitrogen.

### Construction of plasmids and mutants of mAChR

All mutations were introduced into the rat M_3_R [30]. In this rat M_3_R construct the central part of ICL3 was removed and the N- and C terminus contained an HA- and a Flag-tag, respectively. These modifications were previously demonstrated to have no significant effect on receptor function [29-31].

The cDNA from *Photinus pyralis *luciferase (without start and stop codons) was amplified and introduced into the ICL3 of M_3_R by a PCR-based strategy. Luciferase cDNA was 5' and 3' flanked by *Spe*I sites which allowed for systematic and convenient shortening of ICL3 during optimization experiments. All other mutations were introduced by PCR-based and fragment replacement strategies into M_3_R-luci (Figure 1 and 2). For control purposes, several additional constructs were generated. Here, luciferase was integrated into ICL2 (Figure 2, construct #21) or C-terminally fused to M_3_R that was truncated in ICL3 (Figure 2, construct #22). Moreover, luciferase was also integrated into V_2_R lacking the central portion of ICL3 (positions R^243 ^– T^253^). The identity of all constructs and the correctness of all PCR-derived sequences were confirmed by restriction analysis and DNA sequencing.

### COS-7 cell culture and transfection

COS-7 cells were grown in Dulbecco's modified Eagle medium (DMEM, Invitrogen) supplemented with 10% fetal bovine serum (FBS), 100 U/ml penicillin, and 100 μg/ml streptomycin at 37°C in a humidified 7% CO_2 _incubator. Rotifect (Roth) was used for transient transfection. For measurement of luciferase activity, 6 × 10^5 ^cells were seeded into 6-cm dishes and transfected with 2 μg of plasmid DNA. Twenty-four hours after transfection, cells were split into white 96-well culture plates (PerkinElmer) at 30,000 cells per well. IP formation was determined in 12-well plates (1.5 × 10^5 ^cells/well transfected with 0.5 μg of plasmid DNA/well). For the measurement of cell surface expression, cells were seeded into 48-well plates (3.5 × 10^4 ^cells/well) and transfected with 0.2 μg of plasmid DNA/well.

### Functional assays

To measure IP formation transfected COS-7 cells were incubated with 2 μCi/ml of *myo*-^3^H-inositol (18.6 Ci/mmol, PerkinElmer) for 18 h. Thereafter, cells were washed once with serum-free DMEM containing 10 mM LiCl, followed by incubation with ligands for 30 min at 37°C. Intracellular IP levels were determined by anion-exchange chromatography as described previously [37].

The luciferase activity assay was performed with a luciferase activity detection system based on the method described by van Leeuwen et al. [38]. Briefly, after incubation with the ligand in DMEM for 20 min at 37°C, cells were washed with ice-cold PBS. Cell lysis was performed on ice by adding 20 μl of lysis buffer (77 mM K_2_HPO_4_, 23 mM KH_2_PO_4_, 0.2% Triton X-100, 1 mM DTT, pH 7.8) per well. The lysate was mixed with 100 μl of luciferase buffer (20 mM tricine, 2.67 mM MgSO_4_, 0.1 mM EDTA, 33.3 mM DTT, 270 μM coenzyme A, 530 μM ATP, 470 μM d-luciferin, pH 7.8). After 3 min luminescence was measured for 1 sec with a Victor^2^-1420 Multilabel counter (PerkinElmer). Prior to data-collecting functional assays, several tests were performed to ascertain the adequate time for cell lysis and incubation with luciferase buffer. Since both buffers did not contain ligands, diffusion from the receptor was possible. The ligand-induced changes in luciferase activity were stable for at least 10 min after cell lysis [see Additional file 6].

To estimate receptor surface expression, we used an indirect cellular ELISA [39]. Three days after transfection cells were fixed with 4% formaldehyde in PBS for 30 min at room temperature. After washing with PBS and blocking with 10% FBS in DMEM at 37°C for 1 h cells were incubated with biotinylated anti-HA-antibody (Roche, 1 μg/ml in DMEM with 10% FBS) at room temperature for 2 h. Plates were washed and incubated with streptavidin-horse-radish peroxidase conjugate (Roche, 1:5000 dilution in DMEM with 10% FBS) at room temperature for 1 h, followed by extensive washing. Enzymatic reactions were carried out at room temperature in the presence of H_2_O_2 _and o-phenylenediamine. The reaction was stopped by adding 50 μl of 50 mM Na_2_SO_3 _in 1 M HCl. Colour development was measured bichromatically at 492 nm and 620 nm using a Sunrise™ plate reader (Tecan).

A paired, two-tailed Student's t-test was used to detect significant differences in luciferase activity after ligand binding. The number of replicates is given in the respective legends of the figures and table.

## Abbreviations

AU: arbitrary units; FBS: fetal bovine serum; CCh: carbachol; DMEM: Dulbecco's modified Eagle medium; ICL1-3: intracellular loops 1–3; IP: inositol phosphate; M_3_R: M_3 _muscarinic acetylcholine receptor; TMH: transmembrane helix; V_2_R: V_2 _vasopressin receptor.

## Authors' contributions

DT carried out all functional assays and participated in the generation of mutants. DL participated in the generation of the mutants. RS participated in designing the mutants. TS conceived of the study, participated in the design and wrote the manuscript. All authors read and approved the final manuscript

## Supplementary Material

Additional file 1 **Description of control experiments**. This file summarises all control experiments carried out. Click here for file

Additional file 2 **Luciferase can be integrated into other GPCR (e.g. V_2_R) without loosing G protein-coupling abilities**. COS-7 cells were transfected with wild type V_2_R and V_2_R-luci. 48 h after transfection cells were incubated with 100 nM arginine-vasopressin (AVP). Cyclic AMP levels were determined using the non-radioactive cAMP-determination kit (AlphaScreening technology, PerkinElmer). The cAMP level (atmol/cell) of two independent experiments performed in triplicate is given (means ± S.E.M.). Click here for file

Additional file 3 **M_3_R ligands have no effect on soluble luciferase and V_2_R-luciferase fusion protein**. COS-7 cells were transfected with luciferase (A) and V_2_R-luci (B). Increasing concentrations of CCh (square), atropine (open circle), scopolamine (filled circle) and butylscopolamine (diamond) were applied and luciferase activity was determined as described under Methods. The enzyme activities without ligands were 1,905,212 ± 172,463 AU (luciferase), 182,120 ± 18,188 AU (V_2_R-luci). Enzyme activity of the individual constructs without ligands was set 100%. All data are given as mean ± S.E.M. of three independent experiments each performed in triplicate. Click here for file

Additional file 4 **Modulation of enzyme activity in M_3_R-luciferase fusion proteins by different receptor ligands**. COS-7 cells transfected with M_3_R-luci and construct #8 were stimulated with the indicated ligands (100 μM) and luciferase activity was determined. The luminescence of the fusion constructs were 184,314 ± 75,049 AU (M_3_R-luci) and 140,452 ± 55,426 AU (construct #8). Enzyme activity of the individual constructs without ligands were set 100%. All data are given as mean ± S.E.M. of four independent experiments each performed in triplicate. Click here for file

Additional file 5 **Primer used in this study**. This table listes all primers used to generate the mutants of the M_3_R-luciferase fusion protein. Click here for file

Additional file 6 **Kinetics of luciferase activity assay**. Cells were transfected with M_3_R-luci and luciferase and the assay was performed as described in the Method section with the exception that the luciferase buffer was added at different incubation times after lysis. Data are given as mean ± S.D. of one experiment performed in triplicate. Click here for file

## References

[B1] Cabantous S, Terwilliger TC, Waldo GS (2005). Protein tagging and detection with engineered self-assembling fragments of green fluorescent protein. Nat Biotechnol.

[B2] Paulmurugan R, Gambhir SS (2006). An intramolecular folding sensor for imaging estrogen receptor-ligand interactions. Proc Natl Acad Sci USA.

[B3] Ostermeier M (2005). Engineering allosteric protein switches by domain insertion. Protein Eng Des Sel.

[B4] Dueber JE, Yeh BJ, Chak K, Lim WA (2003). Reprogramming control of an allosteric signaling switch through modular recombination. Science.

[B5] Guntas G, Mansell TJ, Kim JR, Ostermeier M (2005). Directed evolution of protein switches and their application to the creation of ligand-binding proteins. Proc Natl Acad Sci USA.

[B6] Gloriam DE, Fredriksson R, Schioth HB (2007). The G protein-coupled receptor subset of the rat genome. BMC Genomics.

[B7] Haitina T, Fredriksson R, Foord SM, Schioth HB, Gloriam DE (2009). The G protein-coupled receptor subset of the dog genome is more similar to that in humans than rodents. BMC Genomics.

[B8] Quignon P, Giraud M, Rimbault M, Lavigne P, Tacher S, Morin E, Retout E, Valin AS, Lindblad-Toh K, Nicolas J, Galibert F (2005). The dog and rat olfactory receptor repertoires. Genome Biol.

[B9] Gilchrist A (2007). Modulating G-protein-coupled receptors: from traditional pharmacology to allosterics. Trends Pharmacol Sci.

[B10] Versele M, Lemaire K, Thevelein JM (2001). Sex and sugar in yeast: two distinct GPCR systems. EMBO Rep.

[B11] Schoneberg T, Schulz A, Gudermann T (2002). The structural basis of G-protein-coupled receptor function and dysfunction in human diseases. Rev Physiol Biochem Pharmacol.

[B12] Altenbach C, Yang K, Farrens DL, Farahbakhsh ZT, Khorana HG, Hubbell WL (1996). Structural features and light-dependent changes in the cytoplasmic interhelical E-F loop region of rhodopsin: a site-directed spin-labeling study. Biochemistry.

[B13] Ghanouni P, Steenhuis JJ, Farrens DL, Kobilka BK (2001). Agonist-induced conformational changes in the G-protein-coupling domain of the beta 2 adrenergic receptor. Proc Natl Acad Sci USA.

[B14] Jensen AD, Guarnieri F, Rasmussen SG, Asmar F, Ballesteros JA, Gether U (2001). Agonist-induced conformational changes at the cytoplasmic side of transmembrane segment 6 in the beta 2 adrenergic receptor mapped by site-selective fluorescent labeling. J Biol Chem.

[B15] Rasmussen SG, Jensen AD, Liapakis G, Ghanouni P, Javitch JA, Gether U (1999). Mutation of a highly conserved aspartic acid in the beta2 adrenergic receptor: constitutive activation, structural instability, and conformational rearrangement of transmembrane segment 6. Mol Pharmacol.

[B16] Ward SD, Hamdan FF, Bloodworth LM, Siddiqui NA, Li JH, Wess J (2006). Use of an in situ disulfide cross-linking strategy to study the dynamic properties of the cytoplasmic end of transmembrane domain VI of the M3 muscarinic acetylcholine receptor. Biochemistry.

[B17] Granier S, Kim S, Shafer AM, Ratnala VR, Fung JJ, Zare RN, Kobilka B (2007). Structure and conformational changes in the C-terminal domain of the beta2-adrenoceptor: insights from fluorescence resonance energy transfer studies. J Biol Chem.

[B18] Li JH, Han SJ, Hamdan FF, Kim SK, Jacobson KA, Bloodworth LM, Zhang X, Wess J (2007). Distinct Structural Changes in a G Protein-coupled Receptor Caused by Different Classes of Agonist Ligands. J Biol Chem.

[B19] Vilardaga JP, Steinmeyer R, Harms GS, Lohse MJ (2005). Molecular basis of inverse agonism in a G protein-coupled receptor. Nat Chem Biol.

[B20] Armbruster BN, Li X, Pausch MH, Herlitze S, Roth BL (2007). Evolving the lock to fit the key to create a family of G protein-coupled receptors potently activated by an inert ligand. Proc Natl Acad Sci USA.

[B21] Coward P, Wada HG, Falk MS, Chan SD, Meng F, Akil H, Conklin BR (1998). Controlling signaling with a specifically designed Gi-coupled receptor. Proc Natl Acad Sci USA.

[B22] Schulein R, Rutz C, Rosenthal W (1996). Membrane targeting and determination of transmembrane topology of the human vasopressin V2 receptor. J Biol Chem.

[B23] Nakano Y, Nystedt S, Shivdasani AA, Strutt H, Thomas C, Ingham PW (2004). Functional domains and sub-cellular distribution of the Hedgehog transducing protein Smoothened in Drosophila. Mech Dev.

[B24] Zeng FY, McLean AJ, Milligan G, Lerner M, Chalmers DT, Behan DP (2003). Ligand specific up-regulation of a Renilla reniformis luciferase-tagged, structurally unstable muscarinic M3 chimeric G protein-coupled receptor. Mol Pharmacol.

[B25] Gales C, Rebois RV, Hogue M, Trieu P, Breit A, Hebert TE, Bouvier M (2005). Real-time monitoring of receptor and G-protein interactions in living cells. Nat Methods.

[B26] Vilardaga JP, Bunemann M, Krasel C, Castro M, Lohse MJ (2003). Measurement of the millisecond activation switch of G protein-coupled receptors in living cells. Nat Biotechnol.

[B27] Angers S, Salahpour A, Joly E, Hilairet S, Chelsky D, Dennis M, Bouvier M (2000). Detection of beta 2-adrenergic receptor dimerization in living cells using bioluminescence resonance energy transfer (BRET). Proc Natl Acad Sci USA.

[B28] Moreau CJ, Dupuis JP, Revilloud J, Arumugam K, Vivaudou M (2008). Coupling ion channels to receptors for biomolecule sensing. Nat Nanotechnol.

[B29] Maggio R, Barbier P, Fornai F, Corsini GU (1996). Functional role of the third cytoplasmic loop in muscarinic receptor dimerization. J Biol Chem.

[B30] Schoneberg T, Liu J, Wess J (1995). Plasma membrane localization and functional rescue of truncated forms of a G protein-coupled receptor. J Biol Chem.

[B31] Zeng FY, Wess J (1999). Identification and molecular characterization of m3 muscarinic receptor dimers. J Biol Chem.

[B32] Wess J, Blin N, Yun J, Schoneberg T, Liu J, Schwartz TW, Hjorth SA, Sandholm Kastrup J (1996). G protein-coupled receptors: structural basis of receptor assemply and G protein recognition. Alfred Benzon Symposium 39: Structure and Function of 7TM Receptors.

[B33] Wang XC, Yang J, Huang W, He L, Yu JT, Lin QS, Li W, Zhou HM (2002). Effects of removal of the N-terminal amino acid residues on the activity and conformation of firefly luciferase. Int J Biochem Cell Biol.

[B34] Conti E, Franks NP, Brick P (1996). Crystal structure of firefly luciferase throws light on a superfamily of adenylate-forming enzymes. Structure.

[B35] Lysikova M, Havlas Z, Tucek S (2001). Interactions between allosteric modulators and 4-DAMP and other antagonists at muscarinic receptors: potential significance of the distance between the N and carboxyl C atoms in the molecules of antagonists. Neurochem Res.

[B36] Spalding TA, Burstein ES (2006). Constitutive activity of muscarinic acetylcholine receptors. J Recept Signal Transduct Res.

[B37] Berridge MJ (1983). Rapid accumulation of inositol trisphosphate reveals that agonists hydrolyse polyphosphoinositides instead of phosphatidylinositol. Biochem J.

[B38] van Leeuwen W, Hagendoorn MJM, Ruttink T, van Poecke R, Plas LHW van der, Krol AR van der (2000). The use of the luciferase reporter system for in planta gene expression studies. Plant Mol Biol Rep.

[B39] Schoneberg T, Sandig V, Wess J, Gudermann T, Schultz G (1997). Reconstitution of mutant V2 vasopressin receptors by adenovirus-mediated gene transfer. Molecular basis and clinical implication. J Clin Invest.

