# Supplementary material for: Luciferase activity under direct ligand-dependent control of a muscarinic acetylcholine receptor
Source: BMC Biotechnol. 2009 May 18;9:46. doi: 10.1186/1472-6750-9-46 (PMC2689208; doi:10.1186/1472-6750-9-46)
Supplement: Additional file 1 — Description of control experiments. This file summarises all control experiments carried out. [file 1472-6750-9-46-S1.pdf]

Control experiments

For control purposes several receptor-enzyme fusion proteins were generated. Integration of luciferase into the ILC2 (construct #21 in Figure 2) led to a complete loss of receptor cell surface expression and signal transduction abilities (Table 1). Further, luciferase was linked to M_3R construct truncated in the ICL3 (construct #22 in Figure 2). Although, the constructs displayed considerable luciferase activities, CCh and atropine had no effect on enzyme activity (Table 1). As an additional control, we introduced the luciferase into the ICL3 of the V2 vasopressin receptor (V_2R). As indicated by a significant increase in cAMP levels following vasopressin (AVP) application [see Additional file 3], V_2R-luci is functionally expressed at the cell surface (one third of wild type E_{max} value). All M_3R ligands had no effect on enzyme activity when COS-7 cells were transfected with luciferase and V_2R-luci [see Additional file 4].
