# Supplementary figures and images for: Luciferase activity under direct ligand-dependent control of a muscarinic acetylcholine receptor
Source: BMC Biotechnol. 2009 May 18;9:46. doi: 10.1186/1472-6750-9-46 (PMC2689208; doi:10.1186/1472-6750-9-46)

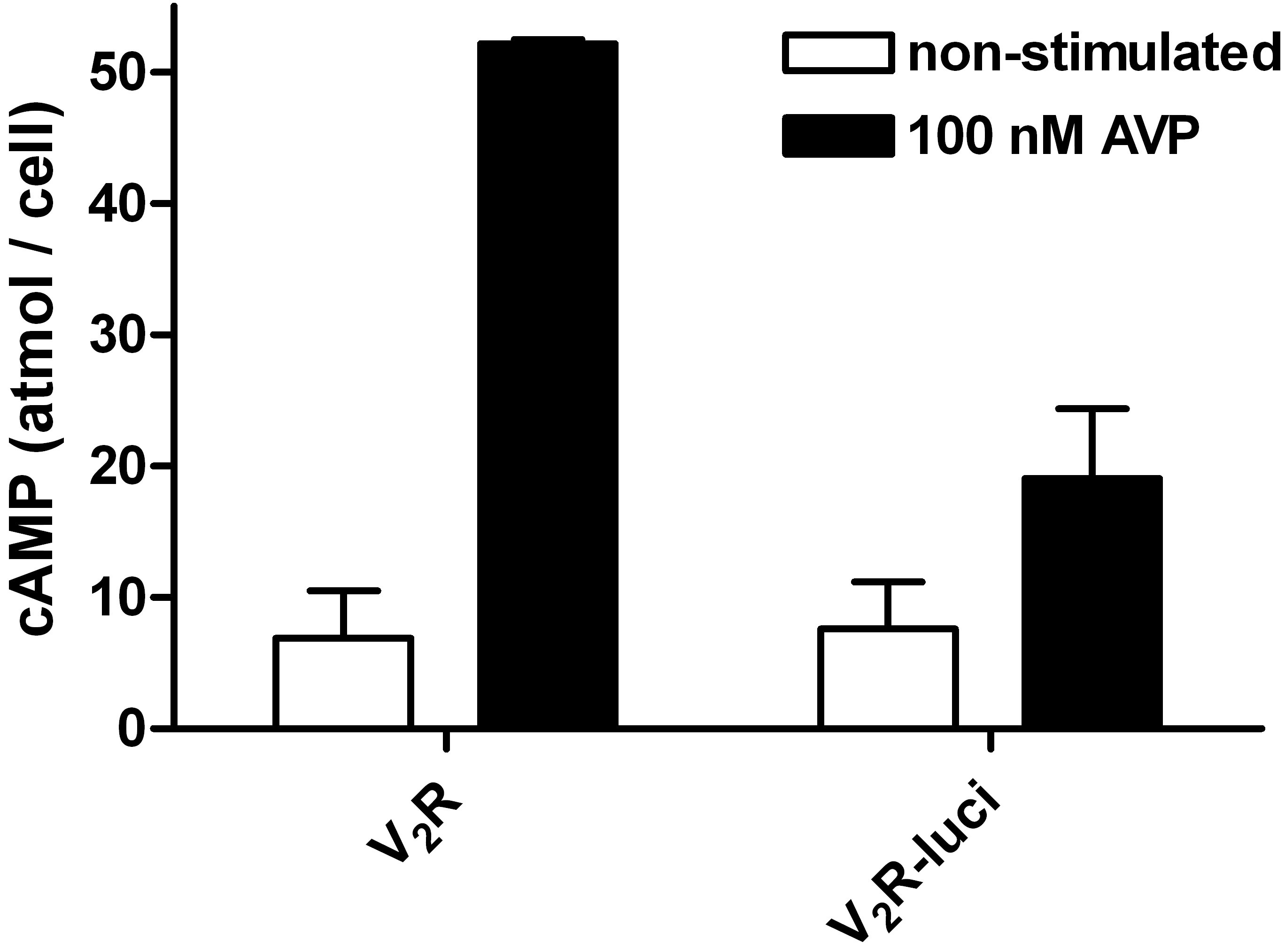

Supplement: Additional file 2 — Luciferase can be integrated into other GPCR (e.g. V2R) without loosing G protein-coupling abilities. COS-7 cells were transfected with wild type V2R and V2R-luci. 48 h after transfection cells were incubated with 100 nM arginine-vasopressin (AVP). Cyclic AMP levels were determined using the non-radioactive cAMP-determination kit (AlphaScreening technology, PerkinElmer). The cAMP level (atmol/cell) of two independent experiments performed in triplicate is given (means ± S.E.M.). [file 1472-6750-9-46-S2.jpeg]

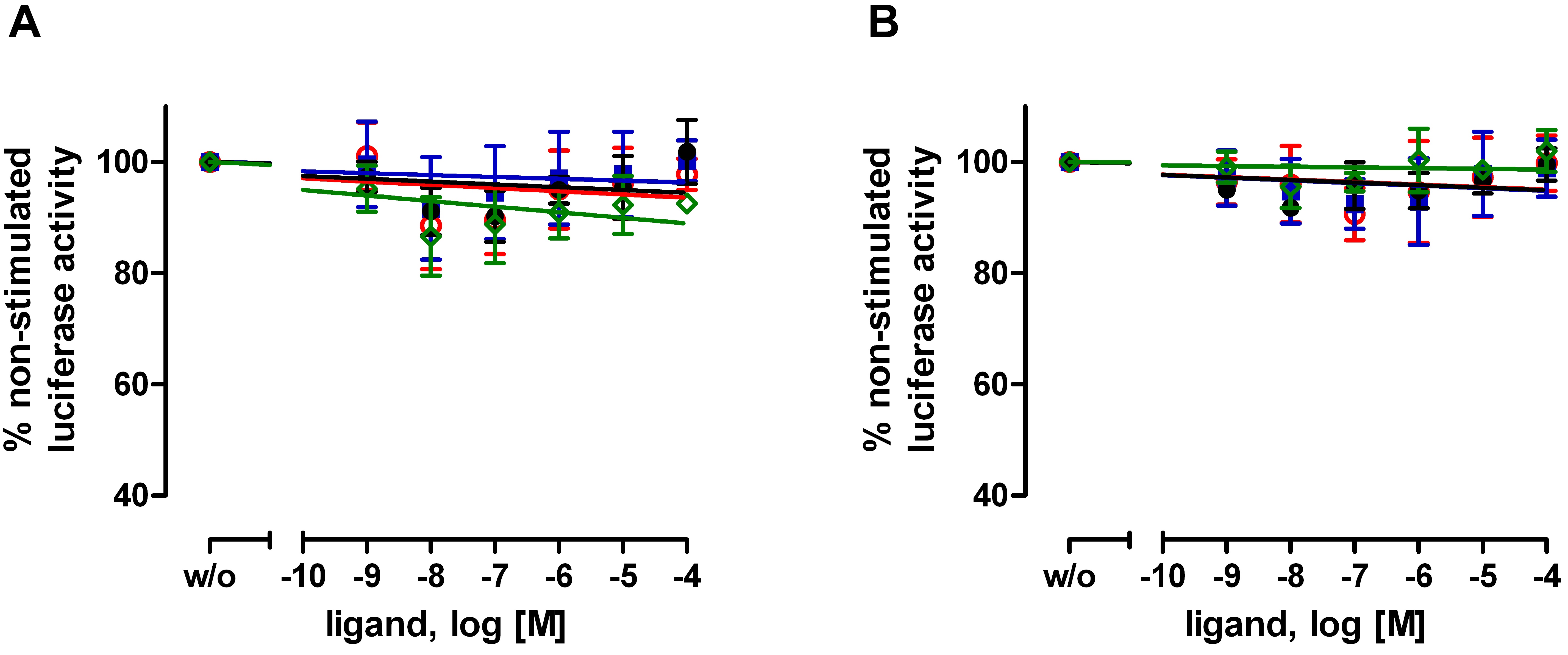

Supplement: Additional file 3 — M3R ligands have no effect on soluble luciferase and V2R-luciferase fusion protein. COS-7 cells were transfected with luciferase (A) and V2R-luci (B). Increasing concentrations of CCh (square), atropine (open circle), scopolamine (filled circle) and butylscopolamine (diamond) were applied and luciferase activity was determined as described under Methods. The enzyme activities without ligands were 1,905,212 ± 172,463 AU (luciferase), 182,120 ± 18,188 AU (V2R-luci). Enzyme activity of the individual constructs without ligands was set 100%. All data are given as mean ± S.E.M. of three independent experiments each performed in triplicate. [file 1472-6750-9-46-S3.jpeg]

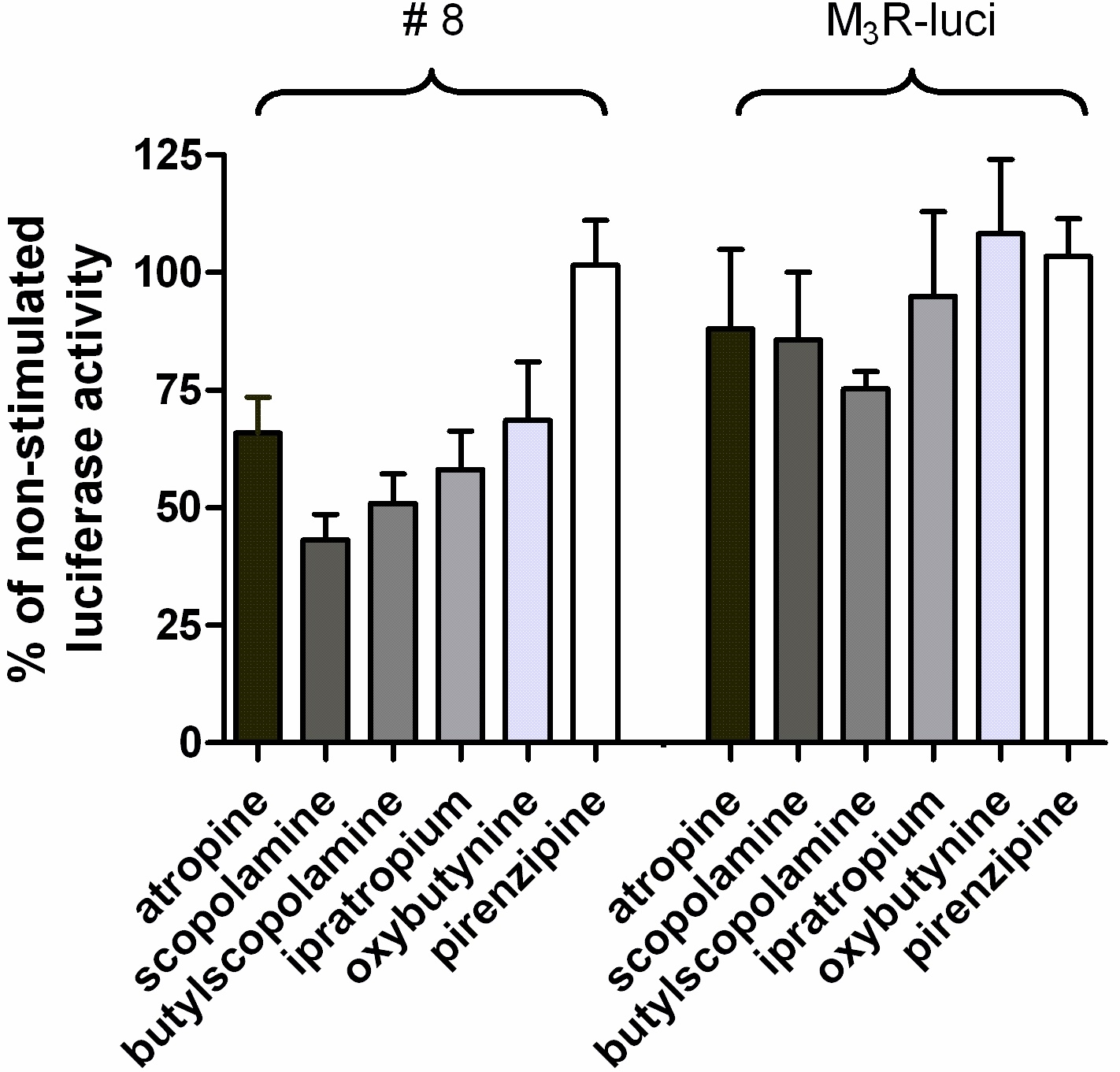

Supplement: Additional file 4 — Modulation of enzyme activity in M3R-luciferase fusion proteins by different receptor ligands. COS-7 cells transfected with M3R-luci and construct #8 were stimulated with the indicated ligands (100 μM) and luciferase activity was determined. The luminescence of the fusion constructs were 184,314 ± 75,049 AU (M3R-luci) and 140,452 ± 55,426 AU (construct #8). Enzyme activity of the individual constructs without ligands were set 100%. All data are given as mean ± S.E.M. of four independent experiments each performed in triplicate. [file 1472-6750-9-46-S4.jpeg]

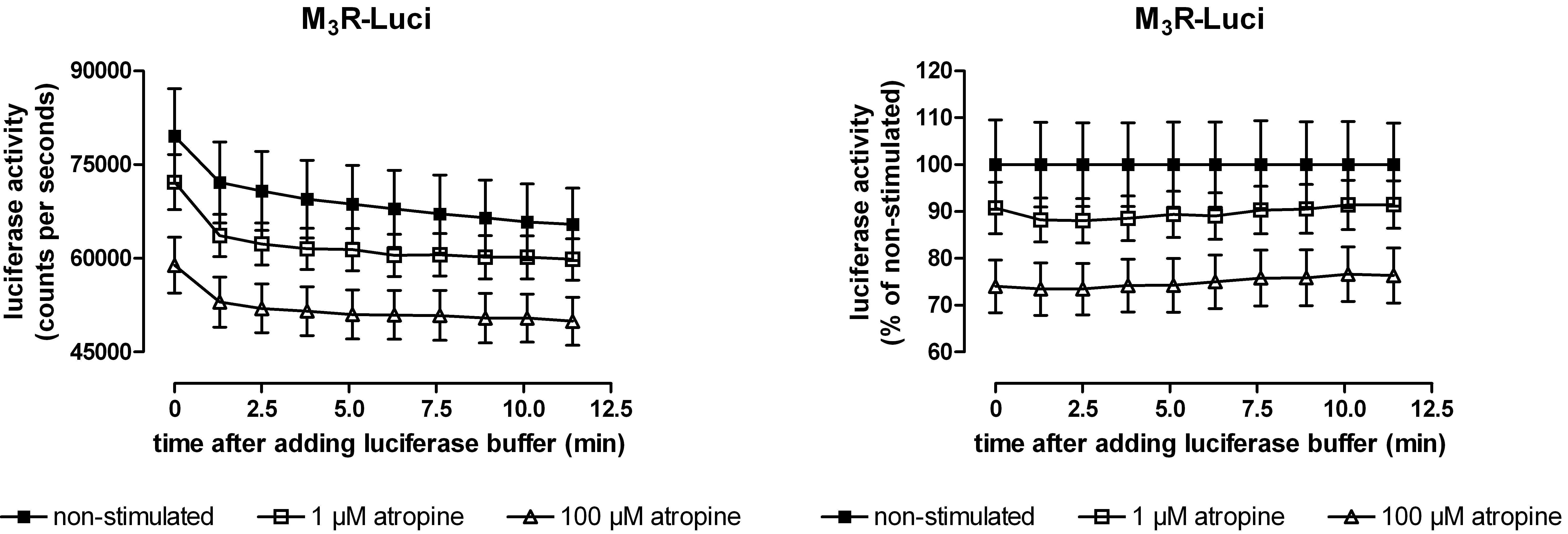

Supplement: Additional file 6 — Kinetics of luciferase activity assay. Cells were transfected with M3R-luci and luciferase and the assay was performed as described in the Method section with the exception that the luciferase buffer was added at different incubation times after lysis. Data are given as mean ± S.D. of one experiment performed in triplicate. [file 1472-6750-9-46-S6.jpeg]
